# Inflammation rapidly modulates the expression of ALDH1A1 (RALDH1) and vimentin in the liver and hepatic macrophages of rats in vivo

**DOI:** 10.1186/1743-7075-11-54

**Published:** 2014-11-25

**Authors:** Kyoko Ito, Reza Zolfaghari, Lei Hao, A Catharine Ross

**Affiliations:** Department of Nutritional Sciences, The Pennsylvania State University, University Park, PA 16802 USA; Graduate Program in Nutrition, The Pennsylvania State University, University Park, PA 16802 USA; Center for Immunology and Infectious Disease, Huck Institutes of the Life Sciences, The Pennsylvania State University, University Park, PA 16802 USA; Huck Institutes for Life Sciences and Department of Nutritional Sciences, Pennsylvania State University, 110 Chandlee Laboratory, University Park, PA 16803 USA

## Abstract

**Background:**

Members of the ALDH1 protein family, known as retinal dehydrogenases (RALDH), produce retinoic acid (RA), a metabolite of vitamin A, and may also oxidize other lipid aldehydes. Of three related ALDH1 genes, ALDH1A1 is most highly expressed in liver. ALDH1A1 is also rapidly gaining importance as a stem cell marker. We hypothesized that ALDH1A1 may have a broad cellular distribution in the liver, and that its expression may be regulated by RA and perturbed by inflammation.

**Methods:**

Studies were conducted in vitamin A-deficient and –adequate rats that were further treated with all-*trans*-RA or lipopolysaccharide (LPS) to induce a state of moderate inflammation. RALDH1A1 expression was determined by quantitative PCR and RALDH1, as well as marker gene expression, was determined by immunocytochemical methods.

**Results:**

Inflammation reduced ALDH1A1 mRNA in whole liver regardless of the level of vitamin A in the diet (*P* < 0.05), while treatment with RA reduced ALDH1A1 expression only in chow-fed rats. ALDH1A1 protein exhibited diffuse staining in hepatocytes, with greater intensity in the periportal region including surrounding bile ducts. Six h after administration of LPS, portal region macrophages were more numerous and some of these cells contained ALDH1A1. Vimentin, which was used as a marker for stellate cells and fibroblasts, was increased by LPS, *P* = 0.011 vs. without LPS, in both ED1 (CD68)-positive macrophages and fibroblastic stellate-like cells in the parenchyma as well as portal regions. Alpha-smooth muscle actin staining was intense around blood vessels, but did not change after LPS or RA, nor overlap with staining for vimentin.

**Conclusions:**

Acute inflammation rapidly downregulates ALDH1A1 expression in whole liver while increasing its expression in periportal macrophages. Changes in ALDH1A1 expression appear to be part of the early acute-phase inflammatory response, which has been shown to alter the expression of other retinoid homeostatic genes. In addition, the rapid strong response of vimentin expression after treatment with LPS suggests that increased vimentin may be a useful marker of early hepatic inflammation.

## Background

The *ALDH1* gene and protein family is comprised of 3 isoforms, ALDH1A1 (*Aldh1a1* in mouse), ALDH1A2, and ALDH1A3 [1–3], each of which is involved in the irreversible oxidative metabolism of the vitamin A metabolite retinal to form all-*trans*-retinoic acid (RA). Due to the activity of these enzymes in retinoid metabolism the ALDH1A1, ALDH1A2, and ALDH1A3 genes and proteins are alternatively known as RALDH1, RALDH2, and RALDH3, respectively. Each gene is expressed in a different tissue-specific pattern in embryonic and adult tissues [4–6]. Besides their role in RA production, the ALDH1 enzymes are known to be capable of metabolizing several aldehydes including acetaldehyde and lipoxygenase-produced reactive oxygen species [1, 3, 4]. Various functions have been proposed for ALDH1, including as a regulator of hepatic gluconeogenesis [7]. Recently, ALDH1A1 has gained attention as a putative marker for cancer stem cells and progenitor cells [1, 8, 9]. Thus, a further understanding its regulation in vivo is crucial. ALDH1A1 has been studied most extensively in the eye [10], but it is known to be expressed more broadly [1–3], including in the fetal and adult liver [3, 5, 11], lung, kidney, spleen, stomach, intestine, brain, heart, muscle and thymus [3, 12–15], and certain cells of the immune system [3–5, 12–14, 16, 17].

Retinal sits at a pivotal juncture in the retinol metabolic pathway, where it can either be reduced to form retinol, which, in turn, can be esterified to form retinyl esters for storage, or it can be oxidized in an irreversible manner to form RA [4, 18], an important regulator of gene transcription through its binding to nuclear RA receptors [18, 19]. Previous studies conducted in mice have shown that RA regulates *Aldh1a1* expression through an RAR-dependent feedback inhibition mechanism [3, 19, 20]. In previous studies, RALDH1 mRNA was lower in both liver and kidney of rats fed vitamin A-deficient diet compared to vitamin A-adequate rats, while the administration of RA to vitamin A-deficient rats for 4 days restored RALDH1 mRNA levels in kidney but not in liver [6]. In contrast, treatment of vitamin A-deficient rats with either RA or retinol suppressed the expression of the RALDH gene in the stomach and intestine [14]. ALDH1A1 mRNA expression was also suppressed in the liver of mice lacking the arylhydrocarbon receptor*, Ahr,* which was attributed to an increased concentration of RA present in the liver of those mice [20]. The proximal region of the human ALDH1A1 promoter contains a functional DNA response element for RARα that was shown to cooperate with C/EBPβ in the expression of the ALDH1A1 gene in liver cells [20]. RA suppressed the expression of C/EBPβ and, as a result, reduced the activity of the promoter [20]. However, although the proximal region of the rat ALDH1A1 promoter has been shown to be essential for expression it apparently is not responsive to RA in kidney cells [21]. Thus, ALDH1A1 gene expression may be regulated differently in various tissues, or in different cells within tissues.

The regulation and localization of ALDH1A1 in the liver under physiological and pathophysiological conditions is still not well understood. ALDH1A1 has been reported to be present in rat hepatic stellate cells (HSC) [22] and hepatocytes [11]. In the current study, we hypothesized that the expression of ALDH1A1 may be regulated not only by RA but also during inflammation, which has not been studied previously. Other retinoid homeostatic genes including retinol-binding protein (RBP4), lecithin:retinol acyltransferase (LRAT), the short-chain dehydrogenase/reductase known as retSDR1/DHRS3 [4], and the cytochrome P450s CYP26A1 and CYP26B1 have all been shown to be significantly perturbed during inflammation [23–29]. In the present study we have investigated whether differences in vitamin A status and acute inflammation alter the hepatic expression of ALDH1A1, and characterized the localization of ALDH1A1 under these physiological and pathophysiological conditions.

## Methods

### Materials

All*-trans-*RA was purchased from Sigma-Aldrich, St. Louis, MO. LPS purified from *Pseudomonas aeruginosa* was obtained from List Biological Laboratories (Campbell, CA). Vitamin A-deficient and adequate purified diets [30] (D13110G and D02080202, respectively) were purchased from Research Diets, Inc., New Brunswick, NJ. The stock chow diet was Purina Laboratory Rodent Diet 5001.

Alkaline phosphatase-conjugated anti-DIG antibody and nitro blue tetrazolium chloride and 5-bromo-4-chloro-3-indolyl-phosphate, toluidine-salt (NBT/BCIP) was purchased from Roche (Indianapolis, IN). VECTASTAIN alkaline phosphatase universal ABC kit AK-5200, VECTASTAIN® Elite ABC kit and VECTOR® red were purchased from Vector Laboratories, Inc. (Burlingame, CA). Rabbit monoclonal antibody to ALDH1A1 (ab52492) and rabbit polyclonal antibody to alpha-smooth muscle (α-SMA, ab5694) were purchased from Abcam Inc. (Cambridge, MA). Mouse monoclonal anti-vimentin antibody was from eBioscience (San Diego, CA) and mouse monoclonal anti-rat CD68 antibody (ED1) from AbD Serotec (Oxford, UK). Tyramide Signal Amplification (TSA)-Plus Fluorescence Palette System® was purchased from PerkinElmer Life and Analytical Sciences (Boston, MA).

### Animals, diets and treatment design

Approval for the use of animals was obtained from the Institutional Animal Use and Care Committee of Pennsylvania State University. Studies were performed either with rats fed a chow diet or a casein-based purified diet that was either vitamin A-adequate (VAA) or vitamin A-deficient (VAD), as described previously [30]. VAA and VAD rats were generated by feeding female Sprague–Dawley rats (Charles River Laboratories, Boston, MA) VAD AIN-93G diet during the lactation period. From weaning to the time of treatment at 8 weeks of age, the offspring were fed either the same diet (VAD group) or switched to the VAA diet containing 4 mg retinol/kg (VAA group). Rats were housed in groups of 2–3 rats of the same sex in a room maintained at 22°C with a 12–12 hour dark–light cycle, with free access to food and water. At 8 weeks of age the rats were divided in four groups of *n* = 4-5/group, with sexes distributed among all 4 groups, and given one of the following treatments: canola oil orally and saline i.p. (placebo control); RA, 1 μg/g body weight (BW) in canola oil given orally; LPS, 50 μg/100 g BW in PBS injected i.p. [24, 27, 29] or both RA and LPS [27, 29], delivered orally and i.p., respectively. Six hours later, rats were euthanized using carbon dioxide asphyxiation. Portions of liver were frozen in liquid nitrogen while a portion from the center of the left lobe was placed in molds in Tissue-Tek® O.C.T. (Sakura Finetek, Tefface, CA) on dry ice. Another portion from the same region was fixed in 4% phosphate-buffered formalin (Fisher Scientific, Waltham, MA). Vitamin A status was determined by measuring plasma retinol concentration using an HPLC method previously reported [31].

### Quantitative reverse transcription PCR (qRT-PCR)

Total RNA was extracted from liver tissue using methods previously described [29] using TRIzol reagent (Life Technologies, Carlsbad, CA). cDNA was synthesized using M-MLV reverse transcriptase (Promega Co., Madison, WI) and qRT-PCR analysis was performed using 2× iQ™ SYBR® Green supermix PCR Master Mix (BioRad, Hercules, CA). Primers were rat ALDH1A1 (NM_022407 or BC061526): 5′-AATCAAGGAAGCTGCAGGAA-3′, 5′-CACCCAGTTCTCGTCCATTT-3′. Rat Vimentin (NM_031140.1): 5′-AATTGCAGGAGCTGAATGAC-3′, 5′-AATGACTGCAGGGTGCTCTC-3′. Primers were tested by agarose gel electrophoresis following RT-PCR reaction to assure the expected transcript sizes. The ratio of mRNA-to-ribosomal 18S RNA was calculated, with the average value of the control group set to 1.0 prior to conducting statistical analysis.

### In situ hybridization

The localization of rat ALDH1A1 mRNA was assessed by in situ hybridization (ISH). A RNA probe for ALDH1A1 was prepared using methods described elsewhere [32]. cDNA was converted with primer pairs 5′-AGCCAAACCAGCAATGTCTT-3′ and 5′-TTCACAACACCTGGGAAACA-3′ (1925 bp). A sense probe was used for the negative control. Briefly, frozen liver section, 6 μm in thickness, were soaked in ice-cold acetone, fixed with 4% paraformaldehyde (Sigma-Aldrich)/PBS for 15 min on ice, then were incubated in 0.1 M triethanolamine (Sigma-Aldrich) buffer for 5 min, after which 0.5% acetic anhydride (Sigma-Aldrich) was added and incubated for 10 min. Prehybridization was performed with 50% formamide (Sigma-Aldrich) /1X SSC at 60°C for 10 min. After stepwise dehydration with serial dilution of ethanol (50, 70, and 100%) the sections were incubated with the probe in hybridization buffer overnight at 42°C. Finally, sections were washed, blocked, and incubated with alkaline phosphatase-conjugated anti-DIG antibody, and then developed with the substrate NBT/BCIP overnight. To suppress endogenous phosphatase activity and reduce background staining, 0.24 mg/mL of levamisole (Sigma-Aldrich), which was applied with the NBT/BCIP solution. Counterstaining was performed with methyl green dye.

*Immunohistochemistry (IHC).* IHC was performed to detect ALDH1A1 protein expression in the liver, using 5-μm thick sections of formalin fixed and paraffin embedded liver sections. Briefly, paraffin was removed and antigen retrieval was performed using citrate buffer (10 mM citric acid, 0.05% Tween 20, pH 6.0) heated up from 95° to 100°C. Sections were soaked in heated citrate-buffer and incubated for 30 min, followed by cooling down for 20 min at room temperature. After rinsing with PBS for 5 min, the sections were stained with the VECTASTAIN® alkaline phosphatase universal ABC kit AK-5200 following the manufacturer’s instructions. A 1:100 dilution of rabbit monoclonal antibody to ALDH1A1 was used for the primary antibody. Blocking buffer without primary antibody was used for a negative control. NBT/BCIP substrate was applied for 30 min. To block endogenous alkaline phosphatase activity, 0.24 mg/mL of levamisole was applied with the NBT/BCIP solution.

IHC was also used to determine the expressions of ED1, a rat macrophage marker [33], vimentin, a stellate cell/fibroblast marker [34, 35], and α-smooth muscle actin (α - SMA), a marker of activated fibroblasts [36]. The procedures used were similar to those described above except that for ED1 we add a step to quench endogenous peroxidase with 0.3% H2O2/PBS for 10 min, and used VECTASTAIN Elite ABC kit with 3,3-diaminobenzidine as substrate. For vimentin and αSMA, we used VECTOR red instead of NBT/BCIP as the substrate, following the protocol of the manufacturer. Vimentin staining area was quantified using ilastik v0.5.12 for signal classification [37] and NIH ImageJ software (http://rsb.info.nih.gov/ij/) for quantification of signal areas. Each section was coded to remove treatment identity and the area of the image occupied by tissue (excluding large blood vessels considered as non-tissue) was determined for each slide, and the area of vimentin-positive staining was determined for the tissue area. Data were calculated for vimentin-stained sections from livers of 9 rats that were not treated with LPS and 8 rats treated with LPS for 6 h. The results were compared by *t*-test as described below.

### Dual fluorescence in situ hybridization and immunohistochemistry

Liver frozen sections 8-μm in thickness were used to perform ISH followed by IHC as described above. The Tyramide Signal Amplification (TSA)-Plus Fluorescence Palette System® was used instead of NBT/BCIP for development. Quenching of endogenous peroxidase was performed as described in the protocol of the TSA-Plus Fluorescence system before the blocking step. The antigen retrieval step was performed by incubation in 20 μg/ml of proteinase K in Tris-EDTA buffer (pH 8.0) for 10 min at 37°C. Blocking buffer was used as described in the protocol of TSA-Plus Fluorescence system.

### Statistical analysis

Results are shown as mean ± SEM. Student’s t-test, or one- or two-way ANOVA was performed with Fisher’s or Holm-Sidak posthoc tests using PRISM (GraphPad, San Diego, CA) or SigmaPlot (SYSTAT Software, Inc., San Jose, CA). When variances were unequal the data were log10 transformed before analysis. *P* ≤0.05 was considered statistically significant.

## Results

### ALDH1A1 mRNA expression is reduced by LPS regardless of vitamin A status

We first confirmed that the major isoform of RALDH expressed in rat liver is ALDH1A1, similar to reports for human liver [13]. Based on cycle threshold values, the relative expression of ALDH1A1 in the liver of chow-fed rats was 100–1000 times greater than ALDH1A2 and 30 times greater than ALDH1A3. We therefore focused on ALDH1A1 in these studies.

**Figure 1:**
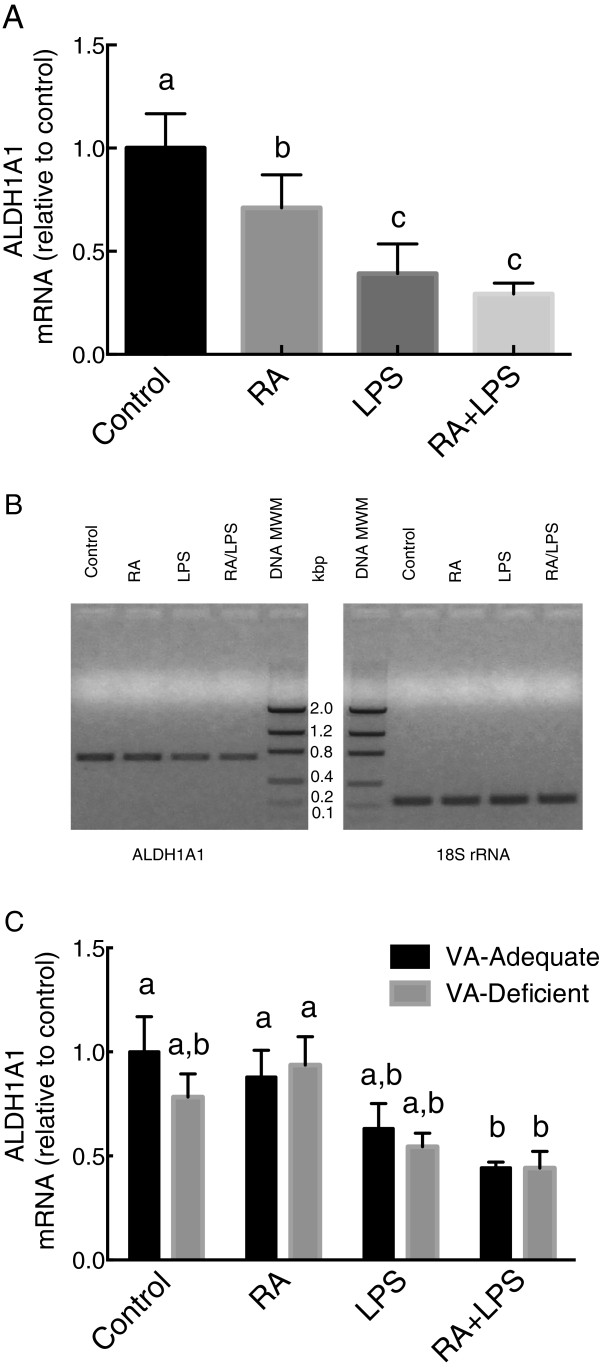
**ALDH1A1 mRNA relative expression levels in 8-wk old rats fed chow diet (A and B) and in VAA and VAD rats (C).** Total RNA was extracted from the liver samples of individual rats and quantified by real time PCR with SYBR Green for ALDH1A1 and 18S ribosomal RNA (rRNA) **(A and C)**. Upon completion, the PCR products from individual samples in Figure 1A were pooled in each group and subjected to ethidium bromide agarose gel electrophoresis, with DNA molecular weight markers (MWM) **(B)**. Values in **A** and **C** were individually normalized to 18S RNA and are expressed as the mean ± SEM of *n* = 4-6/group. Groups not sharing a common letter were significantly different, *P* <0.05 (a > b > c).

In rats fed a normal chow diet, ALDH1A1 mRNA levels were reduced moderately after treatment with RA (*P* < 0.05), while treatment with low-dose LPS [24, 27, 29] for 6 h, as a model for the early stages of mild acute inflammation, resulted in a greater reduction of expression (*P* < 0.05 versus control and RA groups) (Figure 1A), and shown by gel electrophoresis of PCR products in Figure 1B. In rats fed VAA or VAD purified diets, vitamin A status at the end of the study differed significantly as shown by plasma retinol concentration (1.0 μM in VAA vs. 0.2 μM in VAD rats, respectively, *P* < 0.0001). However, there were no differences in body weight, indicating that the vitamin A deficiency was moderate. There were no differences in plasma retinol due to RA or LPS treatment, which may have been due to the short treatment. The relative abundance of ALDH1A1 mRNA did not differ after RA alone. It was reduced marginally but not statistically by LPS treatment in VAA rats (*P* < 0.05), and differed significantly in VAA rats treated with LPS + RA, *P <* 0.05 (Figure 1B). Therefore, both in rats fed chow diet (Figure 1A) and those fed purified diet (Figure 1C), ALDH1A1 mRNA was rapidly and significantly reduced after treatment either with LPS alone (Figure 1A) or with LPS in the presence of RA (Figure 1C).

### Location of ALDH1A1 mRNA by ISH

**Figure 2:**
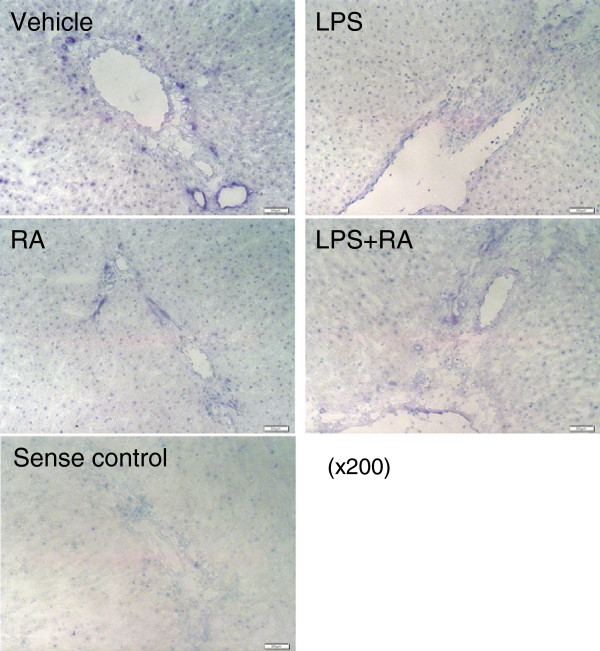
**ALDH1A1 mRNA expression and localization by in situ hybridization.** Livers of VAA rats were used to detect rat ALDH1A1 mRNA expression (purple color). Green signals show nuclei counter stained by methyl green dye. Hepatocytes are notable by their large, round, methyl green-stained nuclei. Staining for ALDH1A1 mRNA was present throughout the parenchyma but was most intense around the portal tracts, including surrounding bile ducts (arrow, vehicle-treated liver). A sense RNA probe control is also illustrated. Magnification: × 200. Results for VAD liver were similar and therefore are not shown.

The localization of ALDH1A1 mRNA in the liver was determined by in situ hybridization (Figure 2). In VAA liver (shown) and VAD liver (similar and therefore not shown) ALDH1A1 mRNA signals were observed throughout the liver with relatively light staining in hepatocytes, which was somewhat weaker surrounding the central vein. Staining using the sense strand control showed only a light and relatively even distribution. Hepatocytes from vehicle-treated rats expressed ALDH1A1 mRNA in or around nuclei, while this was hardly observed in the RA- or LPS-treated groups. ALDH1A1 staining was more intense around vessels, especially in the periportal region, including around bile ducts, as shown in the vehicle-treated section. The sense control showed essentially no discrete staining. Conversely, there was no specific staining around blood vessels in the portal tracts. These results indicate that several types of liver cells express ALDH1A1 mRNA, including vascular epi- or endothelial cells and nonparenchymal cells within the portal tract. Sections from rats treated with LPS, both with and without RA, appeared to be more lightly stained. However, the distribution of ALDH1A1-positive cells was similar.

### Localization of ALDH1A1 protein with the rat macrophage marker ED1 during acute inflammation

**Figure 3:**
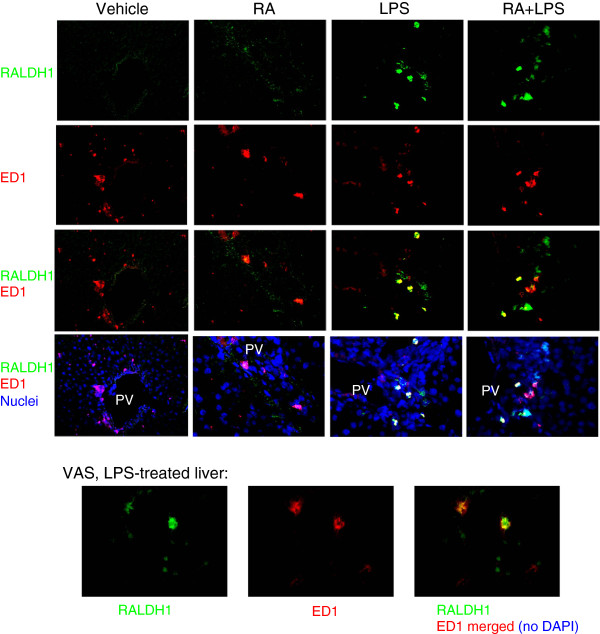
**Dual fluorescence images of ALDH1A1 mRNA with ED1 co-staining for the detection of macrophages.** Dual fluorescence ISH and IHC was performed using rat ALDH1A1 antisense probe and ED1 antibody, a rat macrophage marker, respectively, without and with DAPI staining of nuclei, on sections of VAD rat liver. ED1 was detected in the periportal area. ALDH1A1 staining (green) is increased after treatment with LPS. Some of the cells expressing ALDH1A1 showed overlapping staining with ED1 (red). Yellow; merged signals from ALDH1A1 and ED1; purple, merged signals from ED1 and DAPI nuclear stain. Magnification: x100. The co-localization of ALDH1A1 and ED1 is further illustrated for LPS-treated VAS liver at the bottom of Figure 3, where higher power images are shown (magnification: x200).

Treatment with LPS is well known to induce activation of macrophages [38, 39]. To examine whether macrophages express ALDH1A1, we next conducted dual fluorescence imaging for ALDH1A1 mRNA and ED1 protein, a marker for rat macrophages. Costaining with a fluorescently-stained antisense RNA probe to ALDH1A1 and antibodies to ED1 in rats fed VAD diet (Figure 3), identified some of the cells producing ALDH1A1 (green signals) as being located in the periportal area where ED1 stained macrophages (red signals) were also located. Typical images are illustrated. Although not all macrophages were positive for ALDH1A1, there was still a noticeable increase in ALDH1A1 staining (green) in the LPS and RA + LPS-treated groups compared to the control and RA only groups. Some of the ALDH1A1 signals overlapped with ED1 signals (yellow merged signals, row 3, in the LPS and LPS + RA groups). DAPI staining was used to visualize the nuclei in these sections (row 4). Thus, despite a lower overall expression of ALDH1A1 in the tissue as determined by qRT-PCR (Figure 1), the expression of ALDH1A1 in macrophages was increased by treatment with LPS. The co-localization of ALDH1A1 and ED1 is further illustrated for LPS-treated liver at the bottom of Figure 3, where higher power images are shown. There was a nearly complete overlap of ALDH1A1 and ED1 signals. Together, these results suggest that acute LPS-induced inflammation intensifies ALDH1A1 expression in periportal macrophages.

### Localization of ALDH1A1 protein with the rat stellate cell/fibroblast marker vimentin

**Figure 4:**
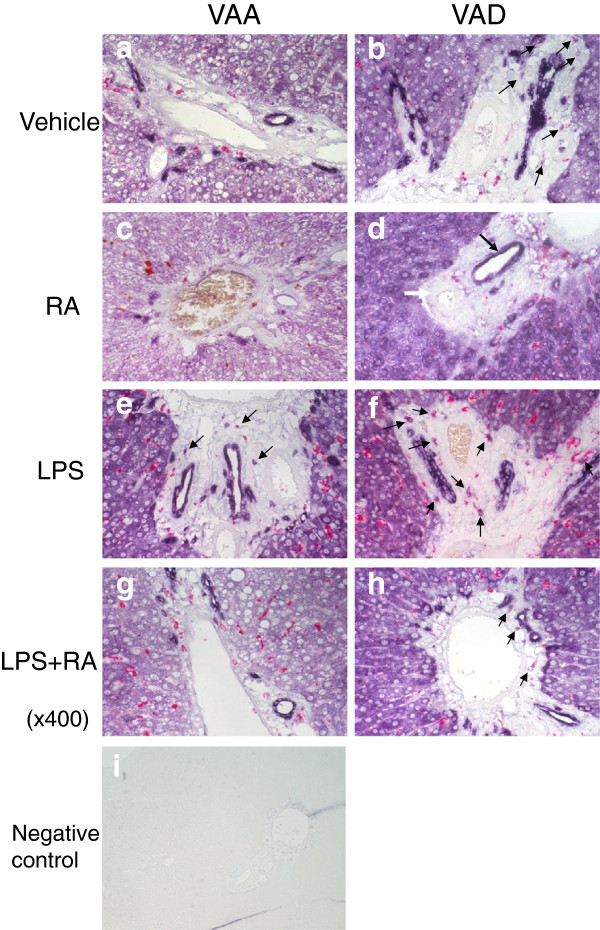
**Co-localization of ALDH1A1 protein expression and rat macrophage marker ED1 in liver from VAA and VAD rats.** Dual IHC with anti-ALDH1A1 antibody and anti-ED1 antibody was performed followed by methyl green counterstaining for detection of nuclei. Anti-ALDH1A1 staining (purple) showed a broad distribution, which was intense around portal regions and bile ducts, while cells staining for ED1 (pink-red) was more generally scattered in the parenchyma and in portal areas after treatment with LPS (Figure 4 **e** and **f** compared with **a** and **b**). Magnification: x400. Panel **i** shows negative staining control.

**Figure 5:**
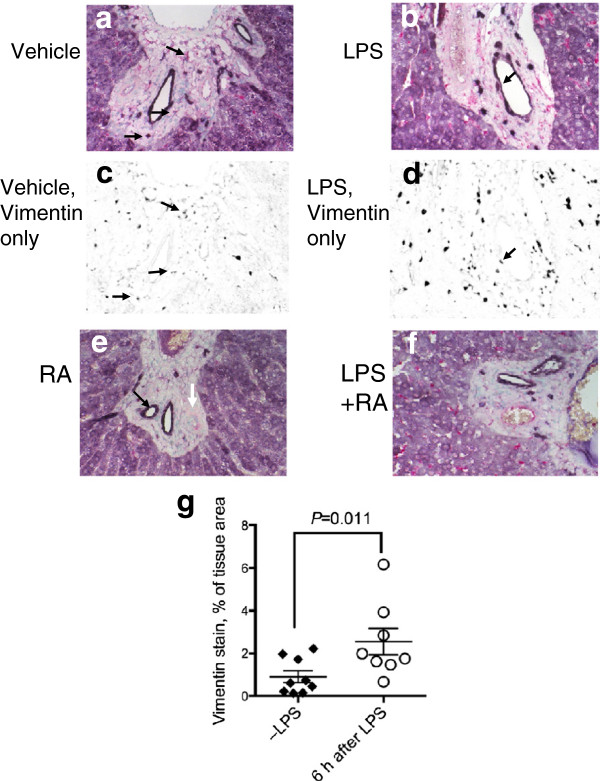
**Co-localization of ALDH1A1 protein expression and stellate cell/fibroblast marker, vimentin in liver from VAA rats.** Results for VAD rats were similar and therefore are not shown. Dual IHC with anti-ALDH1A1 antibody and anti-vimentin antibody was performed followed by methyl green counterstaining for detection of nuclei. Staining controls for vimentin were similar to those shown for ED1 in Figure 4 **i**. Panels c and d show false-color images after ilastik® processing (see Methods) so that only pink (vimentin) signals are visible as black. Arrows illustrate some of the cells that co-stained with purple (ALDH1A1) and vimentin signals (Figure 5 **a-f**). Figure 5e illustrates the intense ALDH1A1 staining around bile duct structures (black arrow) and its absence around the arterial smooth muscle region (white arrow), which is also apparent in other sections. Magnification x 400. Figure 5 **g** shows the tissue area occupied by vimentin staining, analyzed by ilastik®, which was significantly higher in the liver of rats treated with LPS (*n* = 8 animals) compared to those not treated with LPS (*n* = 9 animals, *P* = 0.011).

Additional localization studies with dual IHC using anti-ALDH1A1 and anti-ED1 (Figure 4), or anti-ALDH1A1 and anti-vimentin (Figure 5) were performed on the sections of liver from VAA and VAD rats. We did not observe a noticeable overall decrease in ALDH1A1 protein staining, which may have been due to the short time, just 6 h after the induction of inflammation. Similar to the distribution of ALDH1A1 mRNA, ALDH1A1 protein was present in the parenchyma and in the portal areas, especially surrounding bile ducts where intense staining was observed. ED1 staining (pink signals, Figure 4) was scattered throughout the liver, with more ED1 positive cells both within the portal areas, consistent with Figure 3, and in the nearby parenchyma of LPS-treated VAA and VAD rats. Again, the cells lining bile ducts, but not blood vessels, were stained for ALDH1A1 (seen in several images and marked with black and white arrows, respectively, in Figure 4 panel d). The negative staining control (panel i) was completely clean.

ALDH1A1 protein was also compared with vimentin (Figure 5), used to mark fibroblasts and stellate cells. Results were similar in both VAA and VAD rats and only results for VAA rats are illustrated. In vehicle-treated VAA rats (Figure 5a), vimentin was present mostly within the hepatic portal areas. Some vimentin staining (pink) was also observed around blood vessels from which ALDH1A1 was absent. Vimentin staining was stronger in the portal area and sinusoids of the parenchyma in LPS-treated rats (Figure 5b). Images shown in Figure 5a and b were processed so that only the signals for vimentin are shown in black (Figure 5c, d). These images show that vimentin occupied a greater area in the LPS-treated liver and that vimentin signals were scattered throughout the parenchyma in small irregularly shaped cells, while the hepatocytes that stained positively for ALDH1A1 were relatively free of vimentin. RA-treated rats did not show an appreciable change in vimentin staining (Figure 5c). The staining pattern was similar in RA + LPS-treated rats (Figure 5f) compared to LPS treatment alone (Figure 5b), suggesting that LPS-induced inflammation rather than treatment with RA is mainly responsible for the increase in vimentin protein expression. Vimentin staining was quantified (see Methods) for identically processed liver sections from rats that did not receive LPS (*n* = 9) compared to rats that had received LPS 6 h before tissue collection (*n* = 8), regardless of diet or RA treatment. As shown in Figure 5g, vimentin staining occupied a significantly higher percentage of tissue area in the LPS treated group, *P* = 0.011.

### Lack of colocalization of vimentin and α-smooth muscle actin (α-SMA) in acute liver inflammation

**Figure 6:**
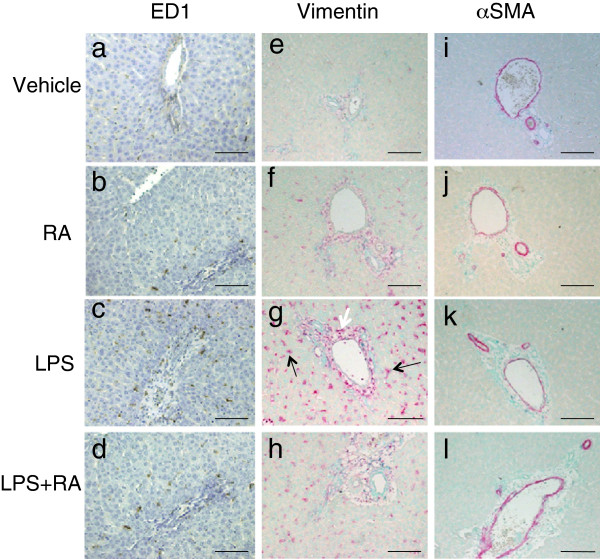
**Detection of macrophages (a-d), vimentin (e-h), and α-SMA (i-l) by IHC in liver of VAD rats.** ED1 staining (brown) detected macrophages; vimentin (red) HSC/fibroblasts, and α-SMA (purple) cells containing smooth-muscle fibers. Nuclei were detected with methyl green dye. Numerous vimentin-positive star-shaped cells are present scattered in the parenchymal after treatment with LPS (panel **g**, black arrows) or LPS + RA (panel **h**). The white arrow in Figure 6g indicates an example of vimentin-positive cells located in the subendothelial mesenchyme. Scale bars; 100 μm.

α-SMA is considered a marker of activated HSC as well as of myocytes. We compared the distributions of staining for ED1, the intermediate filament protein vimentin, and α-SMA after RA, LPS and RA + LPS treatment (Figure 6). Consistent with Figure 3, ED1 staining was more intense after LPS treatment (Figure 6c and d compared to 6a and b). However the change in vimentin staining was more dramatic, shown by a greater number of more intensely stained star- or fibroblastic-shaped cells that were dispersed among hepatocytes, and was most evident in the liver parenchyma of LPS and RA + LPS-treated rats (Figure 6g and h compared to 6e and f). Vimentin-positive cells were also present in the subendothelial mesenchymal tissue around vessels (white arrow in Figure 6g). Vimentin-positive cells were also scattered in the subepithelial mesenchymal tissue of the portal areas (most notable in Figure 6g). On the other hand, α-SMA staining was present only in the smooth muscle cells of vessel walls and not in the underlying connective tissue area, unlike vimentin, and α-SMA staining intensity did not differ appreciably among treatment groups (Figure 6i, j, k, l). Fibroblasts did not stain for α-SMA in our study.

**Figure 7:**
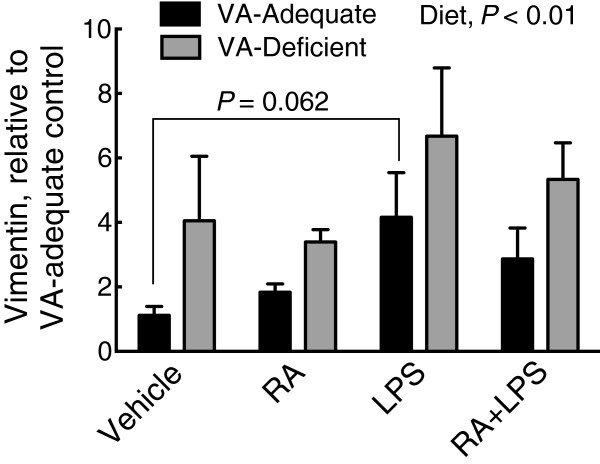
**Relative mRNA expression of vimentin in VAA and VAD diet rat liver.** Vimentin mRNA in VAA (black bars) or VAD (grey bars) rats, determined by qRT-PCR. Normalized values were expressed as the mean ± SEM of *n* = 5 (*n* = 4 for VAD vehicle)/group, with the VAA control group set to 1.0. Diet was a significant main effect, *P* <0.01, and LPS treatment marginally increased vimentin mRNA expression, *P* = 0.062 compared to the VAA vehicle control group.

The increase in vimentin protein observed by histology in Figure 5 was accompanied by a small increase in vimetin mRNA in total liver tissue (Figure 7). Regardless of the treatment group, vimentin mRNA was higher in the liver of VAD rats than VAA rats (diet effect, *P* < 0.01). Average values for vimentin mRNA after LPS treatment were higher in both VAD and VAA groups, consistent with the protein staining results shown in Figure 5g, although this was marginally significant (*P* = 0.062) only in the VAA group.

## Discussion

The exact role and tissue distribution of ALDH1A1 is still unclear, and the effect of inflammation on ALDH1A1 has not been investigated previously. ALDH1A1 has become of increasing interest recently due to its strong association with stem cells [40–42] and as a prognostic cancer marker [43]. However, little is known of its tissue distribution or cellular distribution within tissues under different physiological conditions. In the present study we focused on ALDH1A1 in the liver because it is a major organ of retinoid uptake, storage, metabolism and excretion. We initially observed that ALDH1A1 mRNA is downregulated in the liver of normal, chow-fed rats within a short time, 6 h, after treatment with LPS (Figure 1A), under conditions that have been shown to induce a state of moderate inflammation, characterized by a slight rise in body temperature and lethargy, but from which animals fully recover [24]. Previously, LPS-induced acute inflammation has been shown to modulate the expression of several other retinoid homeostatic genes, notably, the short-chain dehydrogenase-reductase retSDR1/DHRS3, which converts retinal to retinol [28], LRAT, which esterifies retinol [28], and the cytochrome P450 enzymes CYP26A1 and CYP26B1 [29], which oxidizes excess RA [27], all of which were reduced in the liver of LPS-treated rats within 3–6 h after LPS administration [28, 29]. It is reasonable to hypothesize that, if RA is produced by ALDH1A1 in response to a need for RA, then treatment with exogenous RA would result in a down-regulation of ALDH1A1 expression as a feedback system that might effect the gene’s expression [44], while, conversely, animals fed VAD diet and having limited reserves of vitamin A in tissues may have elevated ALDH1A1 expression to compensation for low substrate availability. However, in our study neither a dietary deficiency of vitamin A nor direct administration of RA resulted in a marked change in ALDH1A1 mRNA expression. This result contrasts with an earlier report of reduced ALDH1A1/RALDH1 in the liver of vitamin A-deficient rats, which was not, however, corrected by treatment with RA [6]. Presently, we do not have an explanation for these differences between studies. Inflammation was not studied previously and, in our study, inflammation was a stronger regulator of ALDH1A1 expression than was retinoid status. The physiological role of ALDH1A1 remains uncertain, and the enzyme may metabolize a variety of aldehyde substrates [1, 3, 4, 9]. Our results suggest that their metabolism could be altered in states of inflammation through a reduction in ALDH1A1 expression.

Our study revealed new information on the regional distribution and types of cells that are positive for expression of ALDH1A1. In the parenchymal region, hepatocytes expressed ALDH1A1 and nearly all hepatocytes showed pale to relatively intense cytoplasmic staining. However the most intense staining (Figures 2 and 4) was located in the periportal areas, which are free of hepatocytes but enriched in mesenchymal cells and extracellular matrix proteins [45]. ALDH1A1 staining was also intense in the epithelium surrounding the portal venules and bile ducts (Figure 4a, b). There was little or no staining around blood vessels. In the portal areas, ALDH1A1-stained cells were more intense after treatment with LPS. By fluorescence in situ hybridization, ALDH1A1-positive cells increased in intensity after LPS or RA + LPS treatment and there was overlap of ALDH1A1 expression with at least some of the ED1-positive cells in VAD rats. Thus, changes in the distribution of ALDH1A1 protein were evident very early after induction of inflammation in our model.

Previous reports indicated that ALDH1A1 is expressed in HSC [22] and hepatocytes [11]. HSC are fibroblastic cells with the capability to synthesize retinyl esters from retinol and to store large amounts of retinyl esters in lipid droplets [45–47]. Under normal dietary conditions and when HSC are in the quiescent state [45] about 80% of the liver total vitamin A is stored in these cells [46–48]. However, HSC can become activated by inflammatory stimuli and, during liver injury, undergo a process of activation in which retinyl ester is lost and RA is produced [49] and the cells undergo transdifferentiation in which their stellate-like morphology is lost and the cells become proliferative, contractile, fibrogenic myofibroblasts [45, 49, 50]. This process is considered crucial in the etiology of fibrotic liver disease [51]. Similarly, other mesenchymal cells, such as portal fibroblasts, also can become activated and undergo transdifferentiation into myofibroblasts in the portal area, which may be especially important in biliary fibrosis [45]. Because our studies were of short duration, we would anticipate that we could only observe the very initial changes during the inflammatory, activation process. Thus we can infer that HSC were beginning to become activated by LPS within 6 h, since vimentin, or vinculin (not shown), a focal adhesion complex protein implicated in the migration of activated HSC to sites of injury [52], increased in the liver of LPS-treated rats. At the same time, LPS also resulted in an increase of ALDH1A1 staining in macrophages. These results suggest that inflammation may not only initiate a loss of retinyl esters [49], but also alter the expression of ALDH1A1, which may have a further impact on the liver’s ability to regulate retinoid concentrations. The greater intensity of ALDH1A1 protein in ED1-positive cells suggests that macrophages, like dendritic cells in the intestine [12], may turn on ALDH1A1 expression when they become activated. An interesting question for the future, which our present study did not address, is whether the RALDH1A1-expressing macrophages observed in liver during inflammation are resident macrophages (Kupffer cells), with elevated ALDH1A1 expression, or cells that have infiltrated the liver from the circulation. However, another possible reason for the induction of ALDH1A1 in macrophage after LPS treatment could be that ALDH1A1 is needed to rid cells of substrates other than retinal. As noted in our introduction, the substrate specificity of ALDH1A1 is not yet well clarified. Recently, a role was demonstrated for ALDH1A1 in the clearance of dopamine aldehyde intermediates in neurons [41]. Thus, this enzyme could play diverse and distinct roles in cell biology at different sites. Also recently, ALDH1A1 expression in intestinal CD14+ macrophages in tissue from patients with Crohn’s disease was linked to increased RA production and a more inflammatory phenotype [53].

The intense staining of ALDH1A1 observed in the biliary epithelium was unanticipated and is very interesting. In general, aldehydes are toxic to cells, due to their propensity to react with proteins and lipids to create nonmetabolizable adducts and thus the ALDH1 family of enzymes are in general thought to be protective. In the retina, retinal is known to form potentially toxic products through its reaction with phospholipids and proteins [54]. It is conceivable that ALDH1A1 in the biliary epithelium plays a role in the elimination of excess aldehydes, either retinal and/or other aldehyde compounds, prior to their excretion, and thus prevents their nonspecific reaction with cells of the biliary tract or the small intestine prior to their elimination. The carboxylic acidic products of the ALDH1A1 reaction may also be amenable to further conversion to aqueous-soluble products through glucuronidation or sulfation in phase II metabolic reactions.

Stellate cells are known to be distributed throughout the liver, display a remarkable array of funtions, and to display several patterns of intermediate filament protein expression, including desmin, vimentin, and glial fibrillary acidic protein. The display of various patterns of expression suggests there are subpopulations within this HSC cell type [51]. Activated fibroblasts are known to express α-SMA but the neoexpression of α-SMA in myofibroblasts has been shown to develop slowly, becoming observable after several days during the process of healing of a dermal wound [36]. In our acute studies, we did not observe parenchymal region staining with antibodies to α-SMA, whereas α-SMA staining in blood vessel walls was strong. Stellate cell activation is a more gradual process with respect to loss of retinyl esters stored in lipid droplets and the conversion of cell phenotype from quiescent HSC to the more myofibroblastic phenotype associated with collagen production [45, 47]. In contrast, we observed a rapid change in the level of expression of the intermediate filament protein vimentin (Figure 4B and Figure 5). Vimentin was present in the deeper layers of mesenchyme around the blood vessels and, additionally, in cells with the shape and expected location of HSC scattered throughout the parenchyma (Figure 5). Vimentin has been reported to be expressed in the same mesenchymal cells as α-SMA in some studies, for example in esophageal stromal cells [35], while a study of human fetal liver reported a lack of coincidence of α-SMA and vimentin [55]. The specific role of vimentin is not yet well clarified. As changes in vimentin protein were noticeable in the liver of our rats 6 h after induction of inflammation with LPS, our results suggest that vimentin may be an excellent marker of early hepatic inflammation. Future studies focused on comparing vimentin, α-SMA and retinoid-related genes during both acute and prolonged inflammation, and after acute and chronic liver injury, could help to better understand which types of cells co-express this marker and under which types of physiological and pathophysiological states. The current intense interest in ALDH1A1 as a possible marker of cancer stem cells [1, 8, 9] makes understanding its normal distribution and regulation even more imperative.

## Conclusions

ALDH1A1 was regulated in the early stages of the acute-phase inflammatory response induced by LPS in the intact liver. However, despite the reduction of ALDH1A1 expression in whole liver tissue this was countered by an increased expression in macrophages, suggesting a rebalancing of its expression and possibly of the role of ALDH1A1 during inflammation. Potentially, the increased expression of ALDH1A1 in liver macrophages, which are capable of migration, may help to supply RA in a mobile form that can be transported by these cells to sites of tissue repair or regeneration. These studies add to the understanding of ALDH1A1 expression in the liver and suggest that further functional studies should investigate ALDH1A1 under both normal and inflammatory conditions, with focus on the liver macrophage. In addition, our observations on vimentin expression suggest that this intermediate filament protein may be very useful as a marker of the early stages of HSC activation during inflammation.

## Abbreviations

ALDH: Aldehyde dehydrogenase
HSC: Hepatic stellate cells
IHC: Hmmunohistochemistry
ISH: In situ hybridization
LPS: Lipopolysaccharide
RA: Retinoic acid
RALDH: Retinal dehydrogenase (alterative name for ALDH1)
VAA: Vitamin A-adequate
VAD: Vitamin A-deficient.
